# Dielectric Properties of Aqueous Electrolyte Solutions Confined in Silica Nanopore: Molecular Simulation vs. Continuum-Based Models

**DOI:** 10.3390/membranes12020220

**Published:** 2022-02-14

**Authors:** Haochen Zhu, Bo Hu

**Affiliations:** 1 State Key Laboratory of Pollution Control and Resources Reuse, Key Laboratory of Yangtze River Water Environment, College of Environmental Science and Engineering, Tongji University, Ministry of Education, 1239 Siping Rd., Shanghai 200092, China; 1851535@tongji.edu.cn; 2 Shanghai Institute of Pollution Control and Ecological Security, Shanghai 200092, China

**Keywords:** silica nanopore, dielectric constant, electrolyte aqueous solutions, nanofiltration theory, molecular dynamics simulation

## Abstract

Dielectric behavior of electrolyte aqueous solutions with various concentrations in a cylindrical nanopore of MCM 41 silica has been investigated. The effect of confinement is investigated by using isothermal-isosurface-isobaric statistical ensemble, which has proved to be an effective alternative to the Grand Canonical Monte Carlo (GCMC) simulation method. Several single-salt solutions have been considered (e.g., NaCl, NaI, BaCl_2_, MgCl_2_) in order to investigate the effect of ion polarizability, ion size, and ion charge. The effect of salt concentration has also been addressed by considering NaCl solutions at different concentrations (i.e., 0.1 mol/L, 0.5 mol/L, and 1 mol/L). The motivation in performing this integrated set of simulations is to provide deep insight into the dielectric exclusion in NF theory that plays a significant role in separation processes. It was shown that the dielectric constant increased when ions were added to water inside the nanopore (with respect to the dielectric constant of confined pure water) unlike what was obtained in the bulk phase and this phenomenon was even more pronounced for electrolytes with divalent ions (MgCl_2_ and BaCl_2_). Therefore, our simulations indicate opposite effects of ions on the dielectric constant of free (bulk) and nanoconfined aqueous solutions.

## 1. Introduction

In the nanometric electrolyte solutions, the confined solution contacts with the inner wall of the cavity across a large area and the interfacial solution is affected by the induced electric polarization which determines the intensity of solution-mediated intermolecular forces [1,2]. Meanwhile, the arrangement of water molecules is constrained by the space and modulated by the potential field of microstructure of the cavity wall, resulting in a colorful order degree and phase structure of confined aqueous system [3,4,5]. The local polarization ability of interfacial water molecules also affects the interactions between water molecules and other charged particles, thus affecting many important physicochemical-processes of interfacial water such as surface hydration [6,7,8], ionic solvation [9,10,11,12], and nanofiltration (NF) mass transfer process [13,14]. During the last decade, the main applications in NF have been in water treatment for drinking water production (including ground and surface water) as well as wastewater treatment. However, the mechanism of solute transport (particularly for charged solute) through nanofiltration membranes is not completely understood. The main reason is that the physical behavior involved in the separation process inside a confined membrane is still indistinct. An optimal development of the NF process therefore requires building relevant modelling tools that relate the properties of the membrane material and the fluid confined inside the membrane pores to the separation efficiency. A theoretical work that points out the links between the membrane structure and the transfer properties is thus essential but the complexity of the transport phenomena inside nanometric paths makes this task cumbersome [15,16,17,18,19].

Among which, dielectric exclusion is likely to play a significant role in NF because it is an important indicator of a material’s ability to store electricity [20,21,22]. Therefore, it is of immense importance to grasp the dielectric properties of electrolyte solutions in confined systems. However, the direct measurement of the dielectric constant of confined water is extremely complex and difficult and many researchers thus apply the molecular dynamics (MD) simulation to investigate the exceptional dielectric properties of water confined in the nanometric materials, such as carbon nanotube (CNT) [23,24,25], graphene and its derivates [26,27], and silica nanocavity [28,29].

Dielectric properties confined in a nanoscale environment is forcefully dependent on the structure and morphology of the medium. Because of the nanoconfinement the dielectric constant becomes a function of position inside the enclosure and exhibits anisotropy in a non-homogeneous system. The latter has been deeply investigated in our previous work for pure water inside a silica hydrophilic nanopore and we found that the mean values of the dielectric constants give quite close results, almost independent of the location of the silicon nanopores [29,30,31]. The dielectric constant of confined pure water is reduced by two times, which is due to the strong directivity of the water dipole near the surface, while the water dipole does not show any preferred directivity in the bulk phase. Recently, Fumagalli et al. also verified the radial heterogeneous dielectric constant by the local capacitance measurements between two atomically graphene sheets applying atomic force microscopy. They revealed that it existed as an interfacial layer of imperceptibly tiny polarization [32]. Theoretically, varieties of geometries have been constituted to calculate the local dielectric constant of aqueous solution within cylindrical, spherical, and flat plate confined mediums. Toward this end, Ghoufi et al. has predicted an anomalous variation of dielectric constant of pure water confined in cylindrical and spherical nanoconfinement, respectively [29,33]. These abnormal dielectric variations were also corroborated by Jalali et al. who carried out a series of MD simulations to study dielectric behaviors inside graphene bilayers and demonstrated a non-linear decrease of the dielectric constant inside the confined system [34].

**Figure 1:**
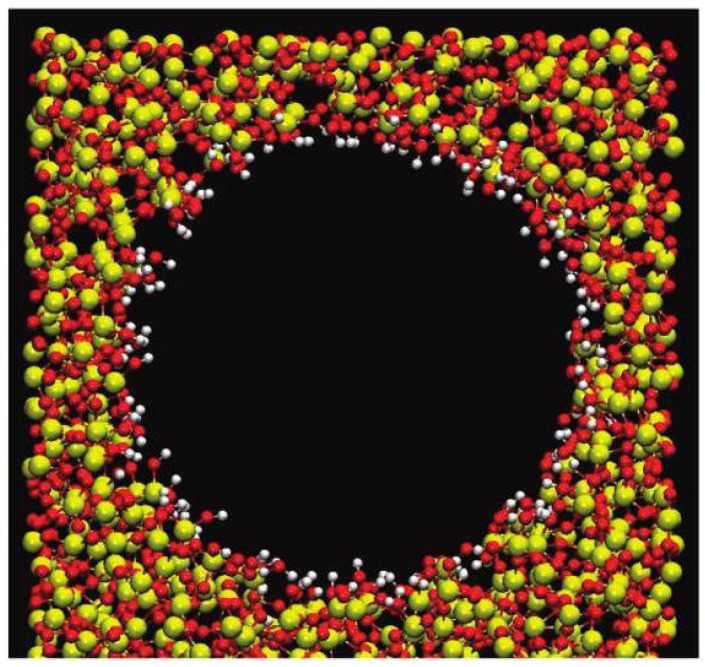
An illustration of MCM-41. Oxygen atoms are in red. The hydrogen positions are in white. Yellow indicates the silicon atoms.

In this research, we have studied the dielectric characteristics of aqueous electrolyte solutions confined in a cylindrical nanopore of MCM 41 silica (see Figure 1) by means of Grand Canonical Monte Carlo (GCMC) simulations with an Isothermal-Isosurface-Isobaric statistical ensemble [35]. Currently, MCM 41, as a kind of nanoporous membrane, has been widely applied due to its high surface area of porous materials that can provide ordered porosity and a high surface area along with strong physical or chemical adsorption [36,37,38,39]. Several single-salt solutions have been investigated (e.g., NaCl, NaI, BaCl_2_, MgCl_2_) in order to study the effect of ion polarizability, ion size, and ion charge. Salt concentration has also been discussed by taking into account different concentrations of NaCl solutions (i.e., 0.1 mol/L, 0.5 mol/L, and 1 mol/L). In order to consider the effect of ion polarization, we have applied the core-shell approach, which accounts fairly well for induced polarization with limited computational cost, given the high explored length and time scales with respect to the induced dipole model and the fluctuation charge model [40]. For liquid/vapor, it has been fully confirmed that the ionic density of the interface region is not affected by the polarizability of water molecules [41]. Thereby, the unpolarized TIP4P/2005 (i.e., Transferable Intermolecular Potential 4/2005) water model was applied, which has had fantastic performance in various physical properties [42].

## 2. Model and Computational Details

**Figure 2:**
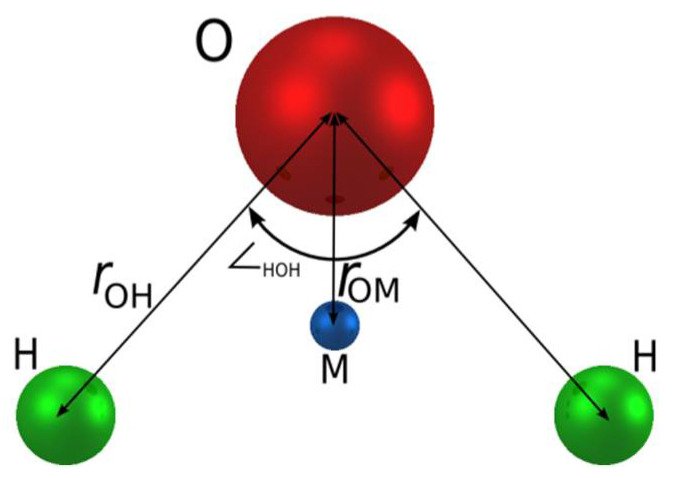
Shape of TIP4P/2005 water model.

The non polarizable TIP4P/2005 water model is a re-parameterization of the original TIP4P [43] potential. This model is a rigid model, based on the Bernal–Fowler geometry and functionality and reproduces most properties of bulk water under ambient conditions (density, vaporization enthalpy, radial distribution function, free energy of hydration, etc.). There are four interaction sites (see Figure 2). Three of them are placed at the oxygen and hydrogen atom positions, respectively. The other site, often called the M site, is coplanar with the O and H sites and is located at the bisector of the H-O-H angle. The geometry of the molecule such as the O-H distance (r_OH_ = 0.9572 Å), O-M distance (r_OM_ = 0.1546 Å) and H-O-H angle (∠__HOH_ = 104.52°) is given by the gas-phase values. Ion polarizability is considered by applying the core-shell model [44] and we show their force field parameters in Table 1.

The most investigated form of silicate MCM-41 matrix was applied to build a nanoporous system, because the geometrical shape of the porous MCM-41 is appropriately characterized on the basis of the channels of a certain section and it has potential to present similar constraints. An atomic characterization of the silicate was processed from a balance configuration of amorphous silica in a cubic cell of 35.5 Å, proposed by Vink and Barkema [45]. Thus, we expected a realistic porosity of the amorphous silica by a procedure devised by Bródka and Zerda [46]. We initially produced a cavity along the *z* axis of the silica unit by wiping off the atoms inside a cylinder with a diameter (D) of 20 Å. According to their coordination numbers, we differentiated bridging oxygens (O_b_) bonded to two silicon atoms from nonbridging oxygens (O_nb_) bonded to only one silicon and bonded to one hydrogen atom (H_nb_). The iterative process of atom disappearance (O and Si) was adopted until only tetra-coordinated silicon atoms in the structure were combined with up to two O_nb_. Eventually, the surface hydroxyl groups were created by saturating the non-bridging oxygens. The hydroxyl group was permitted to rotate around the Si-O bond even though the silicon matrix was set up to be rigid. In reality, this process decreases the atactic inner surface of porous silicates and the interfacial interaction between the matrix and the fluid. On the internal face of the cavity, we set the density of silanol groups as 7.5 nm^−2^, which was considered to be densely hydrated silica (HH MCM-41), and was similar to the surface silica model established by other work [46,47]. The charges and the Lennard–Jones parameters of the different sites are shown in Table 2.

**Figure 3:**
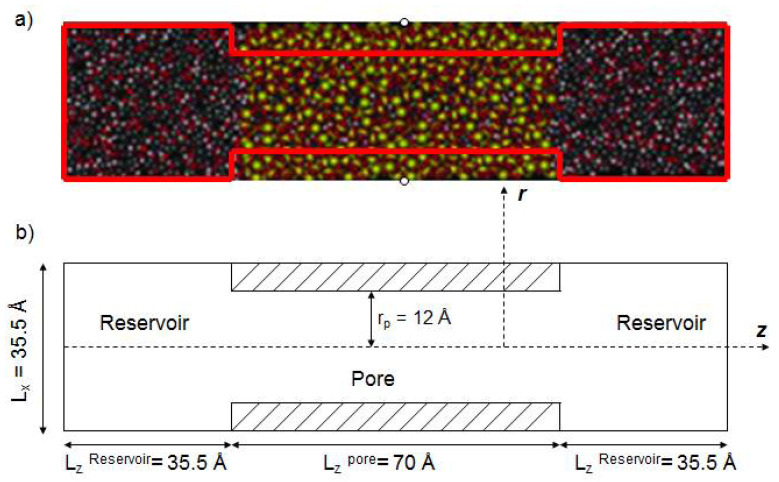
(**a**) Snapshot of the silica nanopore; (**b**) Schematic representation of the silica pore with two reservoirs.

The molecular dynamics simulations of aqueous electrolyte solutions were performed on the isothermal-isosurface-isobaric statistical ensemble (i.e., MD simulations performed in the NP_n_AV_f_T statistical ensemble) with periodic boundary conditions at 298 K. We built our simulation box (see Figure 3) in the x, y, z directions of 35.5 Å, 35.5 Å, 140 Å. All systems studied consisted of 4000 water molecules, 4108 pore atoms and different types and quantities of ions. Table 3 shows the numbers of ions added in the two reservoirs (see Figure 3) and its corresponding salt concentrations. The Lennard–Jones interactions were cut off at 12 Å and the electrostatic interactions were determined by Ewald summation [48]. The velocity Verlet algorithm [49,50,51] was used to integrate the equation of motion with a time step of 2 fs. After the balance of 10 ns, the data of the last 2 ns simulation were analyzed.

## 3. Dielectric Constant Computation

The dielectric constant of a media is a notion which is ruled by the correlation between the macroscopic polarization (P) and the Maxwell electric field inside the media (E) [52]. P is a vector produced in answer to the electric field E which is also a vector and relates to the contribution of dipole moment (M). At the microscopic level, the polarization of the isotropic bulk media (M_bulk_) is in relation to the dipole density and the static dielectric constant ($\varepsilon_{bulk}$) can be calculated by analysing the total dipole moment of water:

(1) $$\varepsilon_{b}=1+\frac{4\pi M^{2}}{3Vk_{B}T}$$

where *V* the sample volume; <⋯> denotes a statistical average over the diverse configurations; *k_B_* is the Boltzmann’s constant. The total dipole moment in the bulk system can be obtained by integrating the local polarization density $\vec{P}\left(r\right)\;$ over the whole system [53],

(2) $$\vec{M}=\int \vec{P}\left(r\right)dr$$

For the solution confined in nano-systems, a local (i.e., spatial dependence) dielectric constant should be considered and calculated from the fluctuation of a dipole for a heterogeneous fluid, which can be derived by the term $\langle \vec{P}\left(r\right).\vec{M}\rangle -\langle \vec{P}\left(r\right)\rangle .\langle \vec{M}\rangle$ [52]. Thus, the dielectric constant of electrolyte solutions ($\varepsilon_{p}$) under confinement (i.e., within the silica nanopore) were computed by this local approach in following expression,

(3) $$\varepsilon_{p}\left(r\right)=1+\frac{4\pi \langle \vec{P}\left(r\right).\vec{M}\rangle -\langle \vec{P}\left(r\right)\rangle .\langle \vec{M}\rangle }{3k_{B}T}$$

## 4. Results and Discussion

**Figure 4:**
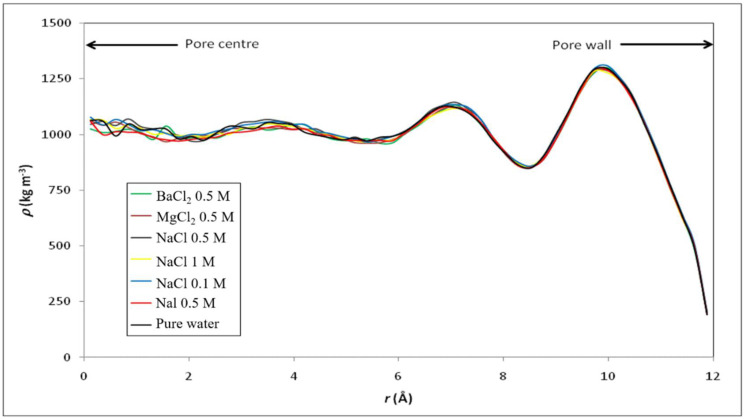
Radial profile of density (*ρ*) for pure water and various electrolyte solutions confined inside the model silica nanopore described in Figure 1.

**Figure 5:**
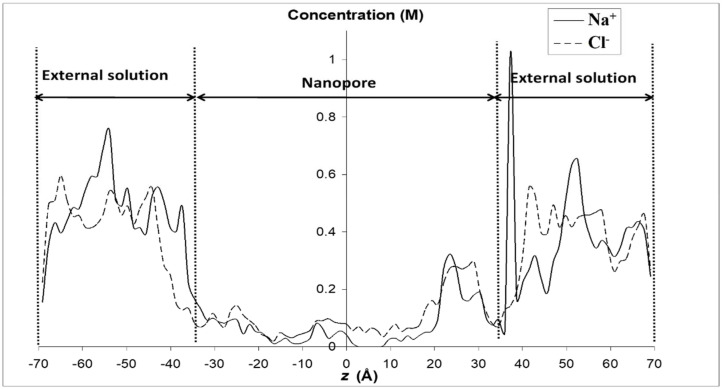
Axial profile of ion density of a 0.5 M NaCl solution brought into contact with the silica nanopore.

We first applied MD simulations to investigate physical properties of various electrolyte solutions confined in the model silica nanopore. However, it should be emphasized that the dielectric constant value of pure water calculated by TIP4P/2005 (~60) is smaller than the experimental value (78). Therefore, we use the ratio to better explore the variation characteristics of dielectric constant inside and outside the nanopore. Our simulation approach through the validation of this water model can be found elsewhere [29]. Figure 4 shows the radial profile of density for pure water and various electrolyte solutions confined inside the silica nanopore. Around the pore center, the density of pure water is close to the bulk one, but some deviations occur closer to the pore surface. The peaks in the density profile observed in Figure 4 indicate a layering of water molecules in the interfacial region. The minimum located at a distance of 3 Å from the surface provides evidence of a region depleted of water in between the two primary hydration layers. The disturbance brought into the density profile by the presence of the pore surface extends up to 8 Å from the pore surface, in keeping with other molecular simulations of aqueous phases in contact with hydrophilic materials like silica [54], platinum [55], or alumina [56]. Such a layering structure of water molecules in the vicinity of a solid surface has been observed with many other solid surfaces and seems to be a general property of any fluid border upon a solid surface [57]. We obtained identical density profiles whatever the electrolyte and its concentration (note that the concentrations indicated in Figure 4 refer to the initial concentrations in the external bulk solutions, ion concentrations inside the nanopore being much lower than the bulk ones as illustrated in Figure 5 which shows the axial ion-density (i.e., along the *z* axis) when the nanopore is brought into contact with a 0.5 M NaCl solution).

**Figure 6:**
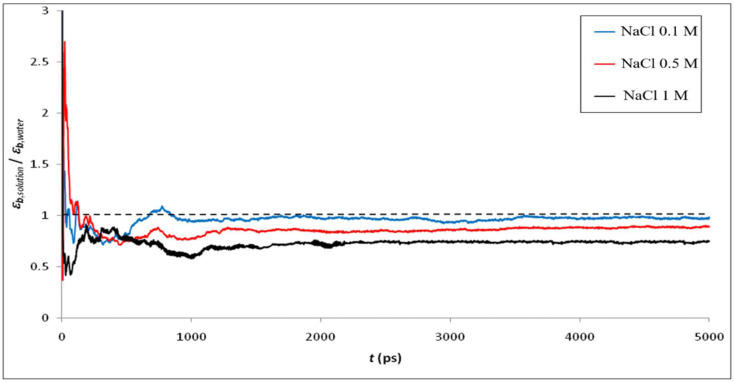
Ratio between the dielectric constant of NaCl solutions of various concentrations and that of water in bulk phase.

**Figure 7:**
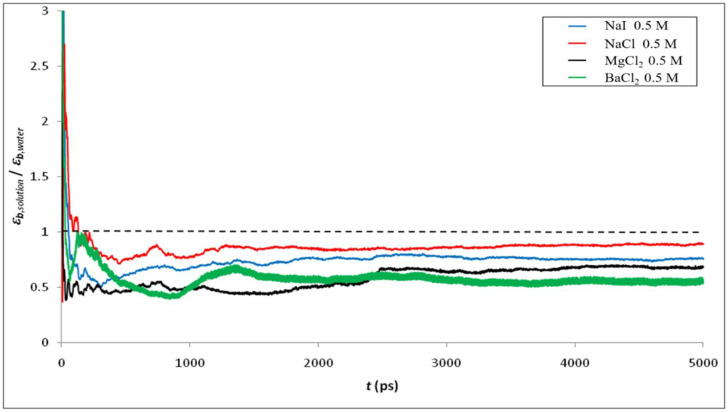
Ratio between the dielectric constant of various electrolyte solutions and that of water in bulk phase.

Figure 6 shows the ratio of permittivity of NaCl solutions with different concentrations calculated by MD simulation to that of bulk phase water. As expected, the addition of ions into water leads to a decrease of the dielectric constant, the effect being more pronounced as the ion concentration increases. Indeed, the electric field generated by ions strongly orientates water molecules in the vicinity of ions, thus limiting the ability of water dipoles to respond to an external electric field [58]. This phenomenon is known as dielectric saturation and is expected to be stronger for multivalent ions than for monovalent ions (due to the larger local electric field around multivalent ions, which affects the orientation of a larger number of surrounding water molecules) as confirmed by our simulations (see Figure 7).

**Figure 8:**
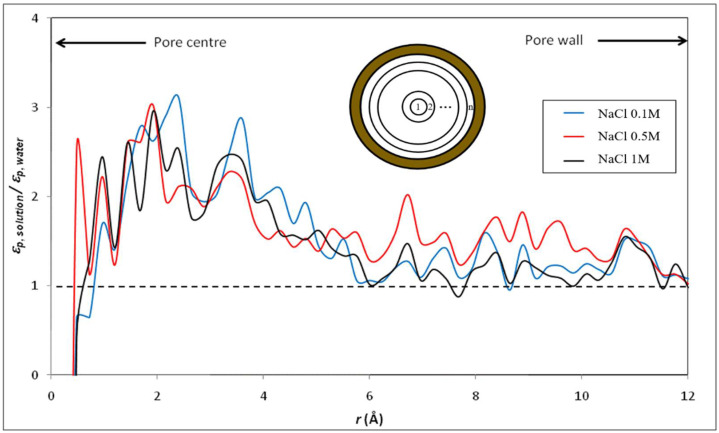
Radial variation of the ratio between the dielectric constant of NaCl solutions of various concentrations and that of water inside the silica nanopore (*n* = 50).

**Figure 9:**
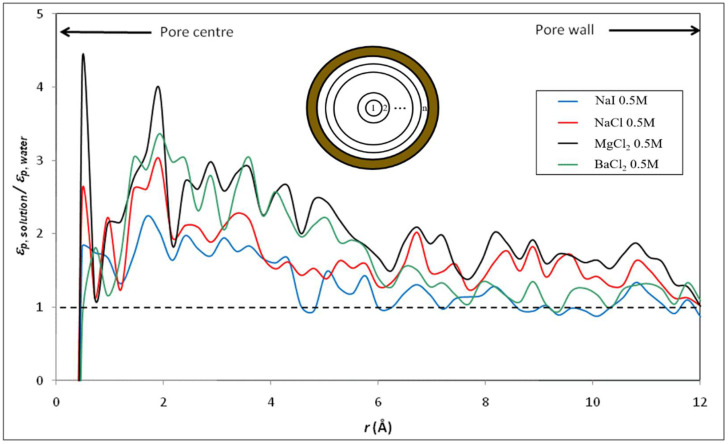
Radial variation of the ratio between the dielectric constant of various electrolyte solutions and that of water inside the silica nanopore (*n* = 50).

**Figure 10:**
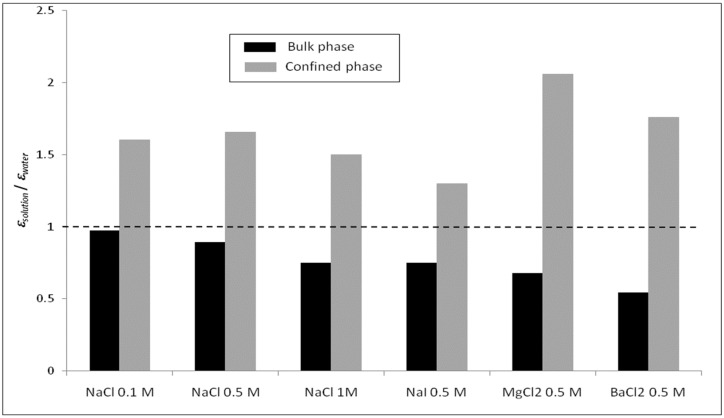
Average ratios between the dielectric constant of electrolyte solutions and that of water in bulk and confined phases.

Furthermore, we focus on the variation of the dielectric constants for the diverse electrolyte solutions in the confined system. Figure 8 and Figure 9 show the radial variation of the ratio between the dielectric constant of various electrolyte solutions ($\varepsilon_{p,\;solution}$) and that of pure water ($\varepsilon_{p,\;water}$) inside the silica nanopore. Note that the calculation of the dielectric constant under confinement depends on the number of water molecules in the MD simulation, which implies that dielectric behavior presents a strong dependence on the size of the nanoconfined system [31]. Thus, the cylindrical nanopores were divided into n concentric shells as illustrated in the inset of Figure 8 and Figure 9. The striking result is that our molecular simulations predict an increase in the dielectric constant when ions are added to water inside the nanopore (with respect to the dielectric constant of confined pure water), unlike what was obtained in bulk phase (see Figure 4 and Figure 5). The phenomenon is even more pronounced for electrolytes with divalent ions (MgCl_2_ and BaCl_2_) as can be seen in Figure 10 which sums up the results obtained in both the bulk and confined phases. Therefore, our simulations indicate opposite effects of ions on the dielectric constant of free (bulk) and nanoconfined aqueous solutions.

**Figure 11:**
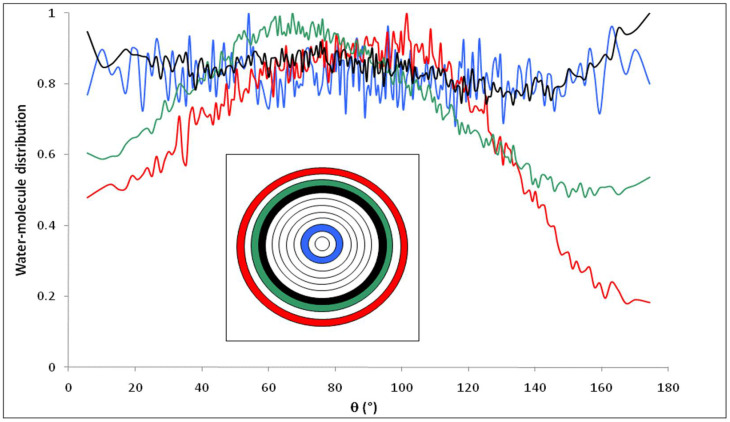
Orientation of water molecules inside the nanopore in contact with 1 M NaCl solution; represents the angle formed by the water dipole moment and the normal vector. The inset shows the location of the different layers inside the pore (*n* = 12).

**Figure 12:**
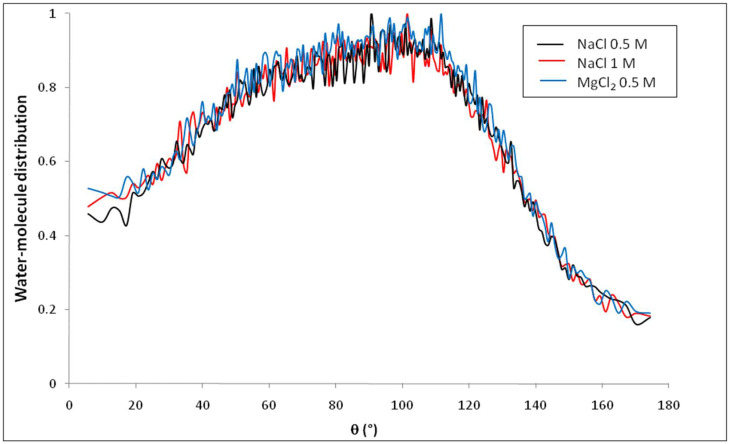
Orientation of water molecules within the first 1 Å thick shell adjacent to the nanopore surface for various electrolyte solutions brought into contact with the nanopore.

Figure 11 shows the distribution of water molecules inside the nanopore as a function of θ, defined as the angle between the dipole moment of a water molecule (${\vec{\mu }}_{H_{2}O}$) and the vector normal to the pore surface ($\vec{N}$) which connects the pore center and the center of mass of the oxygen atom of the water molecule, when the simulation is performed with 1 M NaCl solutions. The pore was divided into concentric shells of 1 Å in thickness. Results show that the presence of ions inside the pore does not affect the orientation of water molecules compared with nanopore that filled with pure water (the distribution of water molecule for pure water has been found superposed with that of NaCl solution inside nanopore). This conclusion is confirmed in Figure 12 which shows that water molecules located in the first interfacial shell keep the same preferential orientation with respect to the pore surface, whatever the added electrolyte.

**Figure 13:**
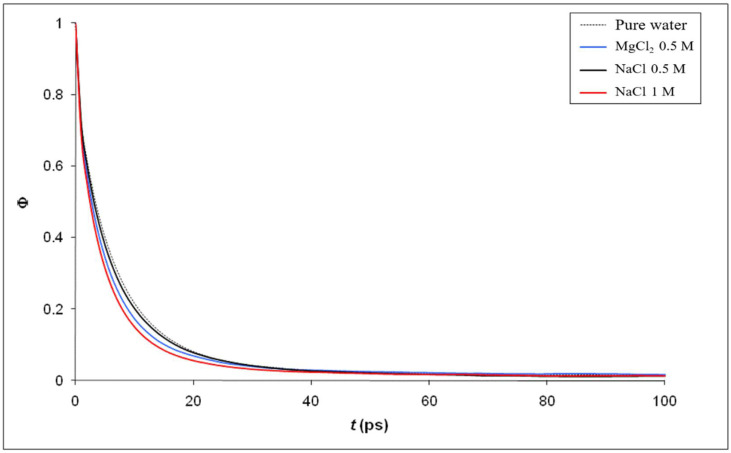
Time correlation function of the dipole moments for pure water and electrolyte solutions confined inside the silica nanopore.

Figure 13 shows the temporal correlation functions of the dipole moments computed inside the nano pore for the same electrolyte solutions, as in Figure 12, and for pure water as well. The autocorrelation function of dipole moments (*Φ*(*t*)) was defined by Guardia and Marti [59]:

(4) $$\Phi \left(t\right)=\frac{\langle \vec{\mu }\left(t\right).\vec{\mu }\left(0\right)\rangle }{\langle \mu {\left(0\right)}^{2}\rangle }$$

and the dynamical dielectric properties of water molecules can be quantified through the correlation time of dipole moments (*τ*) calculated as:

(5) $$\tau =\overset{\infty }{\underset{0}{\int }}\Phi \left(t\right)dt$$

The relaxation time (*τ_rel_*) can be extracted by fitting *Φ*(*t*) with the exponential function [60],

(6) $$exp(-\frac{t}{\tau_{rel}})$$

Interestingly, confined water molecules exhibit a faster relaxation in the presence of salt (the value of τ and *τ_rel_* are collected in Table 4) which might partly explain the results obtained in Figure 6 and Figure 7, i.e., the increase in the dielectric constant inside pores for electrolyte solutions with respect to confined pure water. However, this issue is far from being trivial and deserves a deeper discussion. Most of the remarkable properties of liquid water stem from its ability to form dynamic, labile hydrogen-bond network whose connectivity changes constantly, especially because of the rotation of individual water molecules. Laage and Hynes have proposed a molecular jump mechanism of water reorientation that involves a hydrogen-bond partner switch with a large-amplitude angular jump [61,62]. These authors have also shown that the reorientation amplitude depends on the hydrogen-bond strength [63]. Besides, Mukherjee et al. have investigated the reorientation of water molecules inside very narrow carbon nanotubes and have shown that the reorientational dynamics of confined water molecules is significantly different than that of in the bulk [64]. A better understanding of the results shown in Figure 6, Figure 7 and Figure 8 could come from the study of the reorientation of water molecules in ion hydration shells since recent studies have pointed out that the effect of ions on water dynamics can be strongly interdependent and nonadditive [65]. Notably, investigating the influence of confinement on the molecular mechanism of the reorientation of water molecules in the first and second hydration shells of ions, and thus comparing the rotational degrees of freedom of water molecules surrounding ions inside the nanopore and in the bulk phase, should help us to have a clearer picture of the underlying physics in the results shown in Figure 8, Figure 9 and Figure 10 since the direct link between the static dielectric constant and the dynamics of dipole moments is not obvious (even if it can be noted that Fioretto et al. obtained a qualitatively similar correlation between static dielectric constant and relaxation times in their dielectric relaxation study of water-tert-butanol mixtures [66]).

**Figure 14:**
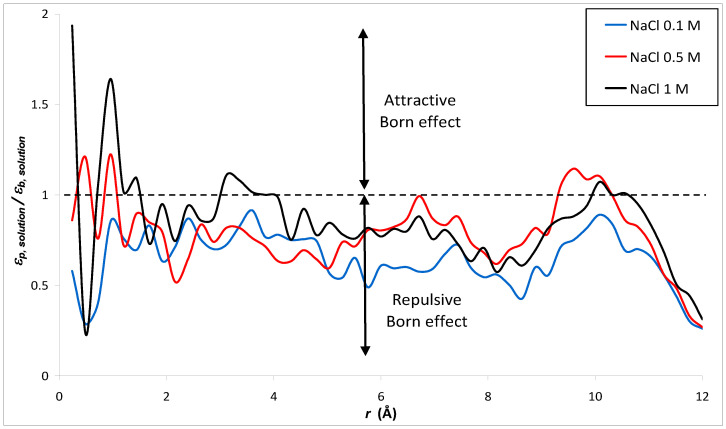
Radial variation of the dielectric constant ratio between confined NaCl solutions and bulk NaCl solutions (*n* = 50). Concentrations indicated in the legend refer to the bulk solutions.

**Figure 15:**
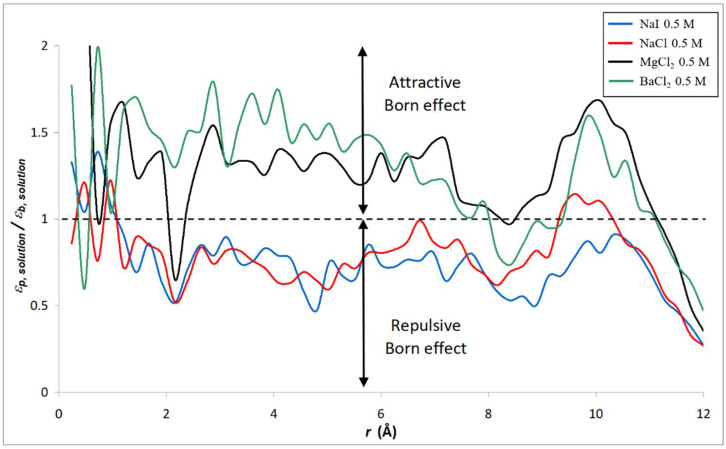
Radial variation of the dielectric constant ratio between confined electrolyte solutions and bulk solutions (*n* = 50). Concentrations indicated in the legend refer to the bulk solutions.

As discussed in the Introduction section, although the necessity to include dielectric exclusion in NF theory is now acknowledged, the dielectric constant of electrolyte solutions inside pores of NF membranes is a fitting parameter in continuum-based models currently used in NF [18,19,67]. The relevance of NF models is currently a matter of debate since in some studies the (fitted) dielectric constant of electrolyte solutions is found to be smaller inside pores than in bulk solutions while other works suggest an increase in the dielectric constant when the electrolyte solution is confined inside the membrane pores. Figure 14 and Figure 15 show the dielectric constant ratio between confined electrolyte solutions and bulk electrolyte solutions, $\varepsilon_{p,\;solution}/\varepsilon_{b,\;solution}$ (note that the concentration indicated in the figure legend refers to the electrolyte concentrations in the bulk phases, concentrations inside pores being smaller due to the nanopore ion-selectivity).

According to the NF theory, a decrease in the dielectric constant of an electrolyte solution inside pores leads to a repulsive Born effect (which in turn increases salt rejection by a membrane) whereas an attractive Born effect arises if $\varepsilon_{p,\;solution}/\varepsilon_{b,\;solution}$ > 1 [67]. As shown in Figure 14 and Figure 15, our simulations lead to a repulsive Born effect for monovalent electrolytes (NaCl and NaI) whatever the bulk concentration while an attractive Born effect is predicted for electrolytes with divalent cations (MgCl_2_ and BaCl_2_). The radially averaged values of $\varepsilon_{p,\;solution}/\varepsilon_{b,\;solution}$ are collected in Table 5. This finding could be of great interest in NF since, to date, model predictions leading to dielectric constants inside pores greater than the bulk values have always been considered as nonphysical results.

Table 6 shows the correlation times of the dipole moments for 0.5 M NaCl, NaI, and MgCl_2_ solutions in the nanopore and in the bulk electrolyte solution. According to Table 6, the repulsive Born effect observed with both the NaCl and NaI solutions might be related to the increase in the correlation time of the dipole moments inside the nanopore. On the other hand, the attractive Born effect shown in Figure 15 for salts with divalent cations like MgCl_2_ might be related to the decrease in the correlation time of the dipole moments inside the nanopore with respect to its bulk value. The same qualitative behavior was obtained with BaCl_2_ (results not shown). It is worth noting that ion concentrations were different inside and outside the nanopore in all simulations performed in this work, due to the selective behavior of the nanopore (as illustrated in Figure 3). As shown in Figure 6 and Figure 7, the effect of ions on the bulk dielectric constant increases with the ion concentration and it is stronger for multivalent ions than for monovalent ones. Therefore, the attractive Born effect obtained with electrolytes having divalent cations could result from the large discrepancy between bulk and pore concentrations. Future work would require the comparison of dielectric constants of both bulk and confined solutions at equal ion concentrations. This could be achieved from simulations of electrolyte solutions confined in an infinite nanopore so as to avoid partitioning effects occurring when an explicit interface is considered between the external bulk solution and the nanopore.

We performed additional MD simulations by considering a negatively charged silica nanopore. The surface charge was generated by removing the protons of all the SiOH groups on the pore surface. The density of SiOH being 7.5 nm^−2^, the fixed charge density ($\sigma_{0}$) of our charged nanopore was about −1.2 C·m^−2^. Although the charge density of NF membranes is expected to be much smaller, the above results obtained with uncharged nanopores can be more easily compared considering that the strongly charged nanopores. All calculations presented below were performed with a molar NaCl solution.

**Figure 16:**
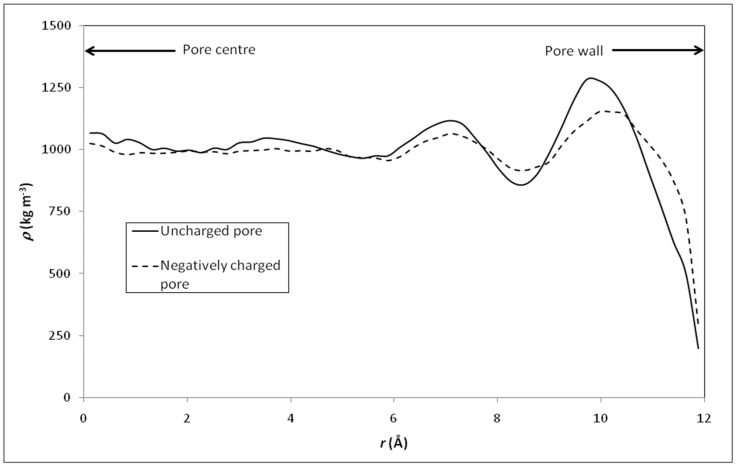
Radial density profile of NaCl solution (bulk concentration: 1 M) inside a negatively charged nanopore (interrupted line) and an uncharged nanopore (full line).

Figure 16 shows the comparison between the radial density profiles of the 1 M NaCl within the negatively charged pore and in the uncharged pore. A layering of the liquid in the interfacial region is obtained for both pores even if the liquid appears to be less structured inside the charged nanopore for which the bulk density is restored at a distance of 6 Å from the pore surface.

**Figure 17:**
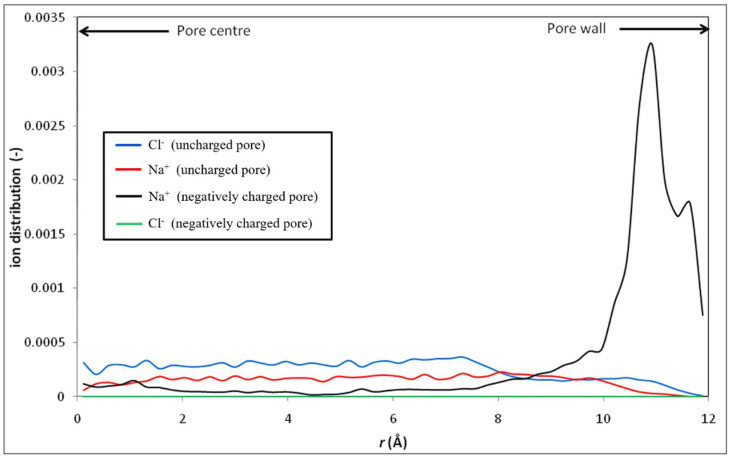
Radial distributions of Na^+^ and Cl^−^ ions inside charged and uncharged nanopores (bulk concentration: 1 M).

Figure 17 shows the radial distribution of Na^+^ and Cl^−^ ions inside both charged and uncharged nanopores. Due to the strong negative surface charge density, the chloride ions are totally expelled from the charged nanopore. Indeed, we did not observe any chloride ions inside the charged nanopore during the simulation run. On the other hand, the sodium cations are attracted towards the surface of the charged nanopore, whereas they stay much farther from the surface of the uncharged pore.

**Figure 18:**
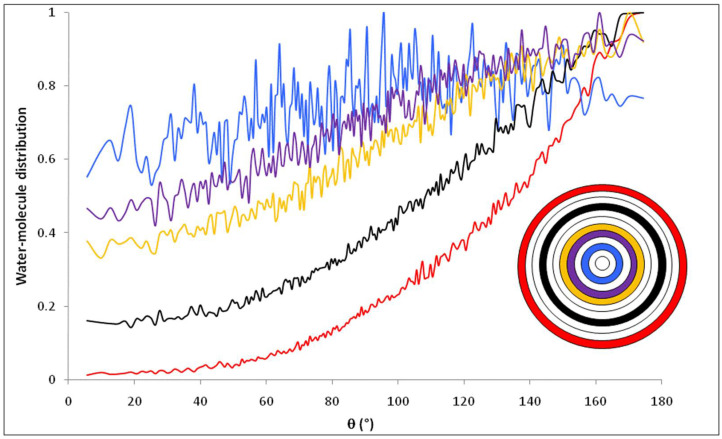
Orientation of water molecules inside the negatively charged nanopore in contact with a molar NaCl solution; represents the angle formed by the water dipole moment and the normal vector. The inset shows the location of the different layers inside the pore (*n* = 12).

Figure 18 shows the distribution of water molecules inside the nanopore as a function of the angle θ between the dipole moment of water molecules and the normal vector to the pore surface. The strong influence of the electric field generated by SiO^−^ surface groups is clearly seen since water molecules located in the first interfacial shell orientate their dipole parallel to the normal vector with θ = 180°. As shown in Figure 16, water molecules keep a preferential orientation over much longer distances from the surface than for the uncharged pore (see Figure 11).

We computed the dielectric constant ratio between the confined and bulk 1 M NaCl solutions for both the uncharged and negatively charged nanopores. Dielectric constant profiles were found to be rather similar for both the uncharged and the charged nanopores and radially averaged ratios were 0.85 and 1.04 for the uncharged and negatively charged pore, respectively. According to statistical uncertainties associated with our MD simulations, it is hazardous to conclude about this apparent discrepancy. Moreover, the simulation time with the charged nanopore was significantly shorter than for the uncharged pore. However, as mentioned previously, the calculation of the dielectric properties requires very long simulation times to converge properly. Therefore, we think that additional simulations should be performed over much longer times so as to be able to conclude about the effect of the surface charge density on the dielectric properties of electrolyte solutions trapped inside nanopores.

## 5. Conclusions

In the present work, dielectric properties of several single-salt solutions (e.g., NaCl, NaI, BaCl_2_, MgCl_2_) confined in a cylindrical MCM 41 silica nanopore have been investigated by means of GCMC simulations. The striking result is that our molecular simulations predict an increase in the dielectric constant when ions are added to water inside the nanopore (with respect to the dielectric constant of confined pure water) unlike what was obtained in the bulk phase and this phenomenon is even more pronounced for electrolytes with divalent ions.

According to the time correlation function, confined water molecules exhibit a faster relaxation in the presence of salt, which might partly explain the results obtained in Figure 6 and Figure 7, i.e., the increase in the dielectric constant inside pores for electrolyte solutions with respect to confined pure water. A better understanding could come from the study of the reorientation of water molecules in ion hydration shells since the effect of ions on water dynamics can be strongly interdependent and nonadditive.

In addition, our simulations lead to a repulsive Born effect for monovalent electrolytes (NaCl and NaI), whatever the bulk concentration, while an attractive Born effect is predicted for electrolytes with divalent cations (MgCl_2_ and BaCl_2_). The repulsive Born effect observed with both NaCl and NaI solutions might be related to the increase in the correlation time of the dipole moments inside the nanopore. On the other hand, the attractive Born effect for salts with divalent cations like MgCl_2_ might be related to the decrease in the correlation time of the dipole moments inside the nanopore with respect to its bulk value. This finding could be of great interest in NF since, to date, model predictions leading to dielectric constants inside pores greater than the bulk values have always been considered as nonphysical results.

Finally, we performed additional MD simulations by considering a negatively charged silica nanopore and computed the dielectric constant ratio between confined and bulk 1 M NaCl solutions for both the uncharged and negatively charged nanopores. Dielectric constant profiles were found to be rather similar for both the uncharged and the charged nanopores and radially averaged ratios were 0.85 and 1.04 for the uncharged and negatively charged pore, respectively.

## Figures and Tables

**Table 1:** Force field parameters for ions [44].

	$q^{ion}(e)$	$q_{D}^{ion}(e)$	σ **(Å)**	ε **kJ·mol^−1^**
Na^+^	+1	−0.6876	2.9234	0.1319
Mg^2+^	+2	−0.4752	2.2528	0.2093
Ba^2+^	+2	−2.1675	3.1435	2.5121
Cl^−^	−1	−3.4572	4.9622	0.3013
I^−^	−1	−4.7331	5.5159	0.8727

**Table 2:** Force field parameters of MCM-41 [35].

	q **(*e*)**	σ **(Å)**	ε **kJ·mol^−1^**
H_nb_	0.206	0.000	0.000
O_b_	−0.6349	2.700	1.622
O_nb_	−0.5399	2.700	1.622
Si	1.2739	0.000	0.000
SiO	0.320	4.500	0.832

**Table 3:** Concentrations and numbers of ions considered in the systems.

	NaCl			NaI	MgCl_2_	BaCl_2_
Concentration (mol/L)	0.1	0.5	1	0.5	0.5	0.5
Number of cation	5	25	50	25	25	25
Number of anion	5	25	50	25	50	50

**Table 4:** Correlation time (*τ*) and relaxation time (*τ_rel_*) of the dipole moments inside the nanopore. Correlation times have been extracted from the first 1 ns of the “exponential” decay of the self-time correlation function of the moment dipoles.

	*τ* (ps)	*τ_rel_* (ps)
Pure water	16.7	6.2
MgCl_2_ 0.5 M	13.7	5.1
NaCl 0.5 M	12.5	5.8
NaCl 1 M	12.7	4.6

**Table 5:** Radially averaged value of $\varepsilon_{p,\;solution}/\varepsilon_{b,\;solution}$ for the various electrolyte solutions considered in Figure 14 and Figure 15.

Electrolyte Solution	$\varepsilon_{p,\;\;solution}/\varepsilon_{b,\;\;solution}$
NaCl 0.1 M	0.65
NaCl 0.5 M	0.78
NaCl 1 M	0.85
NaI 0.5 M	0.74
MgCl_2_ 0.5 M	1.30
BaCl_2_ 0.5 M	1.28

**Table 6:** Correlation time (*τ*) of the dipole moments for confined and bulk solutions. Correlation times have been extracted from the first 1 ns of the “exponential” decay of the self time correlation function of the moment dipoles.

	*τ* (ps)	
	Confined	Bulk
NaCl 0.5 M	12.5	7.6
NaI 0.5 M	14.8	6.4
MgCl_2_ 0.5 M	13.7	25.4

## Data Availability

Data is contained within the article.

## Abbreviations

GCMC	Grand Canonical Monte Carlo
NF	nanofiltration
MD	molecular dynamics
CNT	carbon nanotube
$q^{ion}$	ion charge (*e*)
$q_{D}^{ion}$	ion shell charge (*e*)
$q^{}$	charge (*e*)
σ	the cutoff distance (Å)
ε	the depth of the potential well (kJ·mol^−1^)
P	the correlation between the macroscopic polarization
E	the Maxwell electric field inside the media
M	the contribution of dipole moment
M_bulk_	the polarization of the isotropic bulk media
$\varepsilon_{bulk}$	the static dielectric constant of the isotropic bulk media
V	the sample volume
<⋯>	a statistical average over the diverse configurations
k_B_	the Boltzmann’s constant
$\vec{P}\left(r\right)$	the total dipole moment in bulk system
*ρ*	Radial profile of density
$\varepsilon_{p,\;\;solution}$	the dielectric constant of electrolyte solutions in confinement
$\varepsilon_{p,\;\;water}$	the dielectric constant of pure water in confinement
$\varepsilon_{b,\;\;water}$	the dielectric constant of pure water in bulk phanse
$\varepsilon_{b,\;\;solution}$	the dielectric constant of electrolyte solutions in bulk phase
${\vec{\mu }}_{H_{2}O}$	the dipole moment of a water molecule
$\vec{N}$	the vector normal to the pore surface
Φ(t)	the self correlation function of dipole moments
*τ*	the correlation time of dipole moments
*τ_rel_*	the relaxation time of dipole moments
$\sigma_{0}$	the fixed charge density
